# The Informatics Challenges Facing Biobanks: A Perspective from a United Kingdom Biobanking Network

**DOI:** 10.1089/bio.2014.0099

**Published:** 2015-10-01

**Authors:** Philip R. Quinlan, Martin Groves, Lee B. Jordan, Hilary Stobart, Colin A. Purdie, Alastair M Thompson

**Affiliations:** ^1^ Dundee Cancer Centre, Ninewells Hospital and Medical School, Dundee, United Kingdom.; ^2^ School of Veterinary Medicine and Science, University of Nottingham, Leicestershire, United Kingdom.; ^3^ Computer Science, University of Nottingham, Leicestershire, United Kingdom.; ^4^ Advanced Data Analysis Centre, University of Nottingham, Leicestershire, United Kingdom.; ^5^ NHS Tayside, Ninewells Hospital and Medical School, Dundee, United Kingdom.; ^6^ Independent Cancer Patients Voice, London, United Kingdom.; ^7^ Department of Surgical Oncology, MD Anderson Cancer Centre, Houston, Texas.

## Abstract

The challenges facing biobanks are changing from simple collections of materials to quality-assured fit-for-purpose clinically annotated samples. As a result, informatics awareness and capabilities of a biobank are now intrinsically related to quality. A biobank may be considered a data repository, in the form of raw data (the unprocessed samples), data surrounding the samples (processing and storage conditions), supplementary data (such as clinical annotations), and an increasing ethical requirement for biobanks to have a mechanism for researchers to return their data. The informatics capabilities of a biobank are no longer simply knowing sample locations; instead the capabilities will become a distinguishing factor in the ability of a biobank to provide appropriate samples. There is an increasing requirement for biobanking systems (whether in-house or commercially sourced) to ensure the informatics systems stay apace with the changes being experienced by the biobanking community. In turn, there is a requirement for the biobanks to have a clear informatics policy and directive that is embedded into the wider decision making process. As an example, the Breast Cancer Campaign Tissue Bank in the UK was a collaboration between four individual and diverse biobanks in the UK, and an informatics platform has been developed to address the challenges of running a distributed network. From developing such a system there are key observations about what can or cannot be achieved by informatics in isolation. This article will highlight some of the lessons learned during this development process.

## Introduction

The collection of human material for research is not a new domain, however, it is a domain that is seeing greater attention as the collection of samples and matching data is repeatedly seen as a vital resource in order to facilitate research.^1–4^ The custodians of samples are facing increasing pressures on multiple fronts in order to provide a service that is fit-for-purpose. Sample quality and consistency have received much attention, and there are a series of recommendations on what information should be provided on samples^5–8^ and how analyses of research data should be conducted and reported within journals.^9^

What was clear in the reviews^1,2^ was the importance of matching clinical data for the samples accrued. Indeed, there is some evidence to suggest that there are sufficient samples available in biobanks for translational research, given that only a minority of samples have been used to date in research projects.^10,11^ Contributing factors to the lack of use could be a lack of visibility (i.e., researchers do not know what is available), or that biobanks do not fulfill researcher expectations (such as sample quality or the required clinical annotation).

Gaps remain, and arise, reflecting the evolving requirements from the research community and highlight the importance for any biobank to continually assess the purpose and the focus of the sample and data collection. This customer-focused approach has been described as Biobanking 3.0,^10^ with Biobanking 1.0 described as having the primary focus on the number of samples being collected, and Biobanking 2.0 being a greater focus on quality and data surrounding the specimen (but not necessarily patient data). The shift in emphasis is away from large accrual of samples to more focused collections, specifically ensuring that the data surrounding the sample is of high quality.^10^ Therefore there is an increasing requirement on biobanks to have sufficient informatics capabilities in order to ensure these demands are met.

The International Society for Biological and Environmental Repositories (ISBER) Informatics Survey^12^ found nearly 40% of their respondents were ‘Somewhat or Very Dissatisfied’ with their current information systems. In addition, while Application Programming Interfaces (API) and systems integration were rated as an ‘Important or Very Important’ feature for a biobank database system,^12^ the ability of current database systems to cater to this feature was rated in the lower group of satisfaction. Such a feature is important when attempting to integrate data from multiple sources, including clinical records. To add to this complex picture, 34% of those surveyed used spreadsheets, 36% used paper records for some of their information management, and there was a large mix of labeling techniques used, with 24% still using handwritten tissue storage tubes, which of course impacts how samples can be tracked in electronic systems.

In the UK, the most likely arena for patient recruitment into tissue banks is within the National Health Service (NHS), and as a result the associated clinical data will be extracted from NHS systems. As much as attention is rightly focused on the quality of the sample, attention should also be placed on the quality of the data sources. The first evaluation of data quality within NHS England found areas with potential direct relevance for biobanking initiatives.^13^ Of particular relevance are concerns surrounding the interoperability of clinical systems, the lack of standard terminology to represent medical conditions, and poor attention to the quality of data. Concerns around access to and the quality of clinical data is not solely a problem for the UK. The secondary use of clinical data for research is a concern globally^4^ and clearly poses a challenge to biobanks as downstream users of this data.

The Breast Cancer Campaign Tissue Bank (BCCTB)^14^ was set up in 2010 after a review of the gaps in research infrastructure that highlighted the need for access to high quality and clinically annotated samples.^1^ The BCCTB was specifically initiated to increase the visibility of and access to samples. In the first 5 years of operation, the BCCTB collected samples from four main centers in the UK (the NHS clinical services linked to the Universities of Dundee, Nottingham, Leeds, and the Barts Cancer Institute). The operation of the tissue bank was virtual in nature and despite over-arching standard operating procedures, each center retained operational independence.

Therefore while the BCCTB is a stand-alone biobank, the governance and operational structure was much more akin to a virtualized network. As a result, the challenge was not to set up a new biobank system but to provide a mechanism that could facilitate searching across many existing database systems and structures. Within the University of Dundee, there was an in-house development team that had overseen the development of many tissue bank database systems.^15^ These existing systems were made available to the project at no cost and included the necessary data structures to house the sample and clinical information as well as a tissue request system. Consequently, the team from the University of Dundee provided the in-house support to the BCCTB.

There is always a balance between operating a centralized versus federated search system. The centralized approach has been used^16^ and does offer advantages such as the ability to control data vocabularies and structures (as there is no local freedom), while a disadvantage is that for existing databases there is a necessity to move data between the local and centralized databases. That scenario also undermines the benefits, as the local sites retain the ability to use the vocabularies and structures built into the local database. Federated search systems offer the ability to remotely query databases and therefore reduce the data transfer, such as for federated approaches which have been used previously^17,18^ when integrating biobanks. However, using a federated search does require each database to be equipped with an Application Program Interface (API) to facilitate external queries.

The initial release of the system was built using a federated search system and made available during the launch of BCCTB in early 2011, albeit with only two centers fully integrated into the system. Subsequently with one of the two currently integrated centers changing database systems (with no initial API access) and the other two for a variety of reasons either not having completed the new database installation or insufficient API access on the systems installed, an alternative approach was required. This placed the BCCTB informatics team in the position of having a very similar set of challenges highlighted in the ISBER Informatics Survey:^12^ a range of technical solutions, expertise, and know-how at each center, but seeking to find a solution that could assist in delivering the scientific vision detailed in the gap analysis.^1^ These developments may be widely applicable within the international field of biobanking.

## Methods

The data collection SOP^19^ was created by all centers in response to the gap analysis^1^ and describes the minimum and desirable data required to annotate the samples. The data terms were created in consultation with the member centers after reviewing all the terms used in all centers. Consensus was reached and the informatics team developed the subsequent central structures to store these terms. These data terms were initially created on the assumption that API access would be possible. The BCCTB was, however, in a delicate position, given the reason for the creation of the bank was linked directly to a scientific gap analysis^1^ that identified the need for certain types of samples with a particular level of clinical annotation. However, the banks were not technically equipped to integrate such complex data. The informatics team was therefore re-tasked with attempting to find a solution that could support the local biobanks while still maintaining the ambitions of the network.

**FIG. 1.**
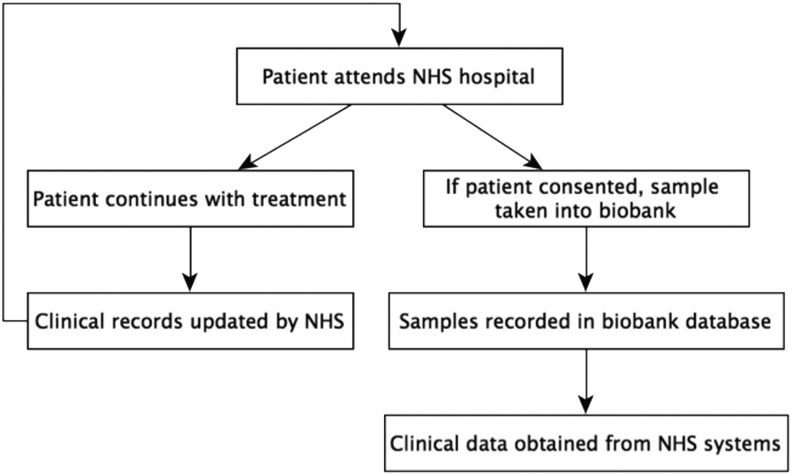
The typical process in the Breast Cancer Campaign Tissue Bank for the accrual of samples and data.

The development goal was to create a single web portal from which researchers could source and request samples from across the network using the terms agreed to in the data standard.^19^ In order to achieve this goal, the web portal must clearly have the ability to identify samples within all member biobanks. The BCCTB followed a mechanism for patient consenting and data accrual, detailed in Figure 1, which will be familiar to many biobanks even if the specific local nuances of the process may vary. The initial dataset surrounding the sample was collected and stored in the local database and at some other point (several months later) further data was secured from the clinical systems to complete the required data set.

These database systems were used to manage their collections but also in varying degrees to access patient clinical information. A network of biobanks is not possible without some form of informatics solution to aid the extraction and/or upload of data from the multiple information management systems and into the central portal. Of course such diversity in information management systems delivers further diversity in other areas, including the data terms used and the respective ability to automate processes, such as exporting data. Each challenge to achieve the goal is described and the solutions to these challenges and the relative success described.

### A wide range of information management systems

To deliver a single web portal from which researchers could source all samples available across the network, there is clearly a requirement to have some form of interoperability between the database systems. In such scenarios there are loosely two options, either perform a federated search (where the central system queries each local system) or require all data to be uploaded centrally. As described, the former was preferred but due to technical limitations at the centers the latter was adopted mid-project.

In this scenario there are effectively two further options: either create one central database that all biobanks are asked to use and where all data should be entered; or create a plugin for each biobank that could provide the necessary functionality to communicate with a central system. The latter was selected as it fit with the model of BCCTB to allow each biobank to retain autonomy within the network and a level of control over the data, but importantly it would provide the necessary capability to run a national network. A software solution was installed in each center (a “Node”), which effectively created a hub and spoke model.

**FIG. 2.**
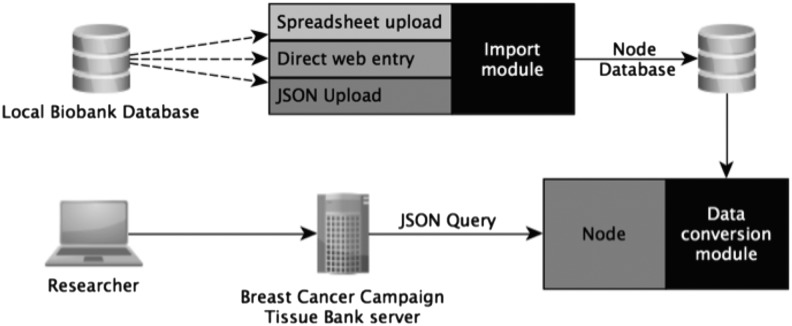
A system process diagram detailing how the biobank can upload data into the Node and how the Breast Cancer Campaign Tissue Bank server interacts with the Node to serve requests from the researcher.

Figure 2 gives an overview of the overall system architecture; all the software developed used open source technology. The database engine was PostgreSQL,^20^ using the DataNucleus^21^ implementation of Java Data Objects and Java^22^ for any scripting. All instances were run on an Apache Tomcat^23^ installation. Each center was given access to an individual instance of the Node, which has the ability to receive communications from the central web portal. The primary role of the Node was to act as a standalone database conduit through which data from the local bank could be made accessible to the wider BCCTB network. Each center would upload their data into the Node to expose it to the BCCTB network. This was an important governance step to ensure each biobank retained control over the data.

In order to cater to the range of systems, the Node accepted data via three methods. The first option was for centers that either used spreadsheets as their database or had a database that could export data into a spreadsheet. The largest challenge in this respect was validating the structure of the spreadsheets. For centers that solely used spreadsheets as their method for collecting and storing data, there is an inherent challenge with how to structure the spreadsheets and how to link data contained within them. For a center choosing to collect data via this method, it would have to create identifiers for each concept (such as a patient, treatment episode, or sample) and also manage the links manually between each concept (i.e., indicating what treatment was for which patient). Not only is this a very laborious process, it is also prone to error. Therefore a core component of the spreadsheet import process within the Node was to check the integrity of the data and to ensure that the data met strict validation rules.

The second option was for direct entry via web interfaces and this was available for centers with no database solution (it could be used as their primary biobank database), but also was appropriate if a site wanted to edit or make corrections on a case-by-case basis. This was an attempt to provide the centers with reduced technical abilities a more enhanced technical solution for their local needs that could be supported by the central informatics team, and in particular to move centers who were using spreadsheets as their primary database onto a more sustainable and sophisticated solution. In particular, using this method meant that centers would no longer have to generate the links between the concepts. The only identifiers the center would have to manage are those for the patient and the identifier on the sample.

The final and preferred option was via JavaScript Object Notation (JSON) as it presented a method for the biobanks to automate the push of data from their biobanks systems into the Node. This approach was preferred as it meant that the staff at the center interacted solely with their local database and never had to deal with the central system. Data updates, identifiers, and terms were automatically handled with no further impact on the staff.

As Figure 2 demonstrates, the central system communicated solely with the Node and in doing so created a unified mechanism for communicating to all participating biobanks, regardless of the underlying database at each center, therefore simplifying communication across the network. By allowing for three types of data entry the system could cater to the large spectrum of database systems and technical abilities at each center.

### Data terms used between the centers

One of the core challenges when bringing together existing biobanks and the associated databases is the array of data standards and data terms that can be used. Each biobank operated within a separate NHS geographical area and therefore interacted with different clinical systems. Given the lack of standard terminology across the NHS, there is an inevitable consequence that the biobanks will be using differing clinical terms. In addition, many of the biobanks had pre-existing collections that would become part of the network and these collections came with pre-existing data terms. While data terms referring to the sample could be adapted for prospective samples, influencing the clinical organization to standardize data terms is a much more ambitious task.

**FIG. 3.**
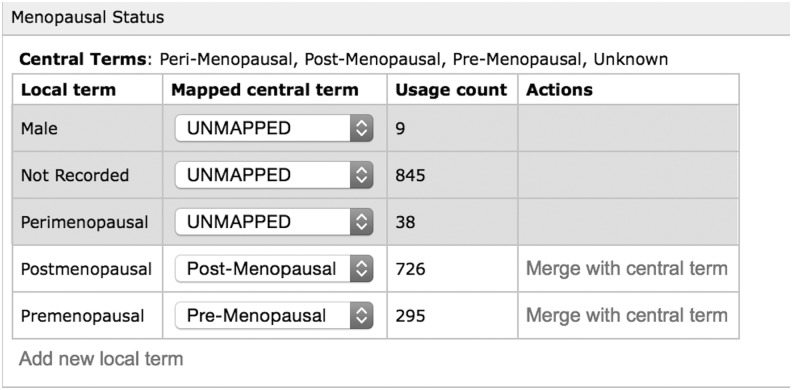
An example of mapping terms for the data field ‘Menopausal Status’. A *row highlighted in red* are terms that are not yet mapped and those in *white* are those that are mapped. The usage count allows the biobank to see how many times this term is used. The biobank can standardize the terms by selecting ‘Merge with central term’, which replace all instances of the local term with the centrally defined term.

The Nodes already provided a level of standardization for the central system and could also be utilized to provide a form of translation between the terms used by the central system and those used at the individual biobank. Within the Node this was known as ‘mapping.’ Each biobank would be presented with a list of terms they have used for a given field and then be asked to provide the necessary mapping to the central term. Figure 3 gives an example of how a biobank can map their terms for menopausal status to the central terms available. In addition to mapping, this also identifies how many times each data term is used and gives them a mechanism to replace their terms with the approved central terms.

The BCCTB central system would only submit a query to the Node using the approved central terms. The Node via the Data Conversion Module (Fig. 2) would then apply any necessary conversions to the data terms in order to run the query using the local biobank terms. Once run, the Data Conversion Module would in turn convert the results back into the approved central terms. Therefore from a central perspective all communications were handled using one centralized data dictionary, despite each center having the ability to use its own terms.

### Aim for the researcher to be presented with a unified view despite differences between centers

**FIG. 4.**
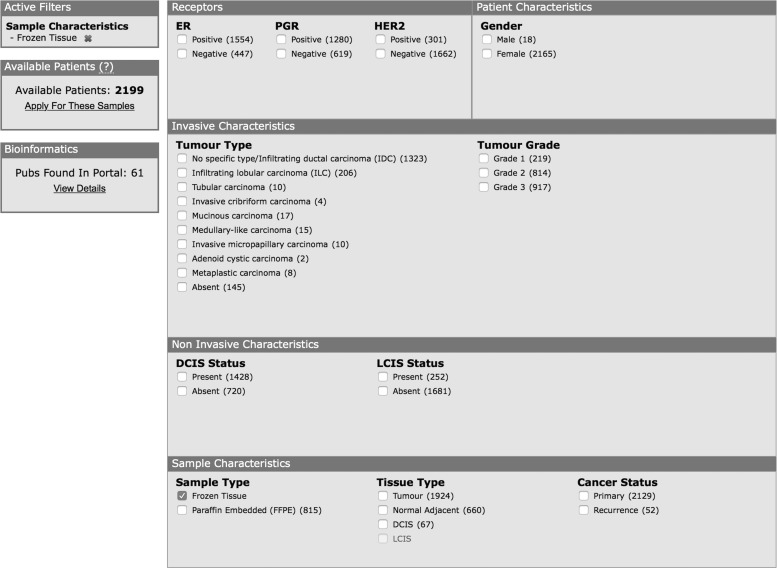
The BCCTB search portal to allow researchers to find samples that are available by clicking on any of the options. In this case, ‘Frozen Tissue’ is selected, revealing 2199 patients with frozen tissue available.

Due to the solutions put in place to address challenges (1) and (2), the researcher was presented with a unified search and tissue request system and the ability to search across all member biobanks using these standard terms, oblivious to the differences that may well exist at each biobank. Figure 4 shows the BCCTB search system^24^ that allows a researcher to find samples in a standardized format, despite the fact that each biobank could be using different terms to represent ‘Frozen Tissue’.

A problem of running a search over multiple databases is the time that queries can take to run, or the possibility that one of the Nodes may be unavailable. The solution adopted was for the central server to hold a cached version of the results, and at a specified time interval the central system would query each Node to receive the latest version of the data. This data returned was a minimal subset to ensure queries could be performed. The mapping and caching process was also adopted for communicating with other external databases such as the Bioinformatics portal (BCCTBbp).^25^

Despite the BCCTBbp using different terms than the central system, the mapping system was used to query the BCCTBbp and store the relevant results in the central cache. As researchers search for samples, they are also presented with the relevant results within the bioinformatics portal. The preference was to have the cache updated daily, so although using a cache does introduce a risk that search data will be slightly out of date (by a maximum of 24 hours), it does remove the need to have a reliable, simultaneous link between all the systems each time a researcher performs a search. Although the initial search is cached, whenever an application is made to BCCTB a full live query is performed to ensure sufficient samples do indeed exist.

## Results

Overall, the three challenges, examined solely from a high level technical perspective, were successfully met. There had been no mechanism to share data between all the member biobanks, no mechanism to handle differences in data terms, and no way for a researcher to be presented with a unified view. The result allowed a rapid process of pseudo-harmonization that would not have been possible without this solution. There was, however, a difference in experience and ease of use reported by different biobanks.

The biobanks with more capable information management systems (using the JSON upload process) found the interaction with the BCCTB system easier than those who relied on uploading spreadsheets (particularly spreadsheets with little input control or structure). As an example, the most advanced solution involved the local biobank system being adapted so that whenever a change was made to the local database, it would automatically send the update to the Node via JSON. The staff at this center had no additional process to perform other than using the local database system. In contrast, those using only spreadsheets to store data had to perform periodic updates (preferably each week), but also retain and manage the necessary identifiers to link the data—a far more complex task with potential to introduce errors.

Examining the process in slightly more detail, the upload of only the sample-derived data was generally perceived well, and as standard terminology was incorporated into prospective collections the process was facilitated. In part this was because as the processes became more standardized, it became easier for those using spreadsheets to upload data.

The real challenge was around the clinical data that was extracted from NHS systems. These systems are generally out of the control of the individual biobank or the informatics team behind the BCCTB system. Therefore, while changes can be made to the biobank processes to forge a level of harmonization across the network to aid the interaction with the central system, this was harder to achieve for the clinical data coming from diverse NHS clinical systems. The BCCTB system propelled the network to a level where the member biobanks as a whole (including upstream NHS systems) were not always comfortable or willing to cooperate. Therefore while the BCCTB system significantly improved the ability of the biobanks to participate and share their samples and data within the network, there was little movement towards overall harmonization of policies, processes, and local informatics solutions across the network. The result of this lack of harmonization is that some centers found using the BCCTB system challenging, as local differences dictated the ease of integration with the BCCTB system.

A disappointing consequence was the inability to move centers from spreadsheets onto the streamlined Node web-based entry. Although this would have reduced the complexity for each center, the preferred method of data capture and entry remained spreadsheets. This was in part because it proved challenging for the central informatics team to develop interfaces and support for centers with great variances in local process. Local network policies, particular on the NHS networks, also restricted access to the Nodes, meaning data could not be entered directly into the Node and the only mechanism was to capture the data in a spreadsheet and subsequently upload. In addition, the central informatics team was not structured to provide resources to the network to assist with change management and user engagement within the biobanks, as the assumption was the local databases would have sufficient API access.

Ultimately, researchers *did* have access to a wealth of samples and clinical annotation that would not have been possible without the Node system. It did provide a mechanism for samples that would have been previously invisible to the network to become available to researchers and thus was a success. However, this does highlight some of the core challenges that will face the biobanking community in the future as the need for virtualized networks increases.

## Discussion

The field of biobanking is changing, with greater focus on harmonization towards networks of integrated biobanks. The BCCTB brought together four UK biobanks to deliver high quality breast cancer samples to the research community based on the perceived gaps in the current infrastructure.^1,2^ The biobanks in the network were representative of the wider field in terms of the array of biobanking capabilities demonstrated in the (ISBER) Informatics Survey^12^ and therefore there was significant diversity in the informatics systems and capabilities at each member biobank.

The informatics team developed a novel solution that could be adapted for many uses beyond breast cancer and indeed beyond cancer biobanks. The system was highlighted as a possible solution for respiratory disease biobanks in the Strategic Tissue Repository Alliances Through Unified Methods (STRATUM).^26^ The data standards developed fed into the development of a new data standard by the Confederation of Cancer Biobanks,^27^ which in many ways is an extension to the MIABIS standard.^5^ Any such system, however, will always be constrained by the storage mechanism, format and structure of data that comes from the information management systems used by the biobanks.

The controlled vocabulary used within the central system was an attempt to harmonize data terms where possible, rather than intended to develop a new ontology for breast cancer. Technical solutions can, and do, exist for integrating biobanks together into a unified network.^17^ The experiences of the BCCTB have shown that the success of implementing such a system is often dictated by a chain reaction of processes that initiates within the environment where the sample is collected. When there is a large disparity between local processes, it becomes increasingly difficult to integrate these under one over-arching informatics solution. To achieve harmonization, the member banks of any network must have a sufficient level of commonality, informatics capabilities, and willingness to integrate with such systems including the upstream clinical systems.

The informatics capabilities of biobanks will increasingly come under scrutiny, for accreditation and regulation schemes, but also from researchers who are becoming more aware of the importance of understanding in much greater detail the characteristics of the patients^5,9^ from whom the samples have been donated. Therefore, access to well-annotated and time relevant samples does remain a challenge. However, the reaction should not be to simply collect more samples. While reports such as the gap analysis^1,2^ focus greater attention on the need for access to high quality and clinically annotated samples, all ten of the key gaps rely on informatics capabilities of one sort or another. Yet neither access to clinical data nor the technical capabilities of biobanks is identified as a current gap, reflecting the underlying significance but as yet unrecognized importance of these elements.

Biobanks have engaged clinical teams for the collection of tissue samples and now this must be extended to the clinical database and informatics systems that will underpin the ability of the biobank to provide the samples required. In addition, the clinical teams need to appreciate the potential benefit the data within the systems could bring to research and work with biobanks to deliver an integrated system. Without such an approach, it is hard to see how biobanks will be able to meet the demands of the researchers and the expectations of the public who have donated their samples for use in research.

Within the UK there is a clear move to improve the quality of data for research use and other downstream collectors of data from the NHS, such as cancer registries, are seeing continually improving data completeness results.^28^ The UK should have an advantage over many countries as it largely operates under one unified health system. Therefore, clearly the ideal system would be one that had seamless integration between the clinical and biobanking systems, potentially incorporating samples as part of the national clinical record. Some have begun to examine the informatics requirements of the clinical care team and the subsequent secondary use for research in the one over-arching informatics strategy,^29^ and in particular, basing it on a common ontology for both clinical and research applications.^30^

The BCCTB has demonstrated the significant challenges that face the developer of any virtualized network when integrating biobanks with poor technical capabilities and/or biobank information management systems. Therefore, while the previous guidance still holds regarding the approach to take and in particular to ensure the network operates at a level that all members are comfortable,^17^ there is a need for the wider community to consider how to respond to the demands of researchers for much richer sample annotation. From this demand comes a clear need to move away from catering to the lowest common denominator and to give at least equal attention to the quality of the information management systems used by the biobanks as is given to the quality of the sample. There is a well-accepted position that samples should only be collected if they are of sufficient quality; however, the quality of the sample is irrelevant if the sample is not used. While the informatics capabilities of biobanks remain consistent with what is represented within the ISBER informatics survey,^12^ forging virtual networks of biobanks with meaningful annotation will be extremely challenging.

Any project seeking to integrate data from multiple biobanks will always be constrained by the technical capabilities of the biobanks within the network. If the aim is to integrate biobanks into national and international networks to ensure the maximum exposure and use of samples, then there are three associated objectives.

The first is to undertake a similar gap analysis^12^ but rather than focusing on scientific gaps to focus on technical gaps that will prevent the scientific vision from being realized. This full assessment should seek to establish if the informatics capabilities exist both in the clinical environment from which the patient is consented, and the biobank. This technical gap analysis will ensure there is a full understanding about how an integrated network can be achieved, both in terms of the initial technical build of the system but also the subsequent support required for the individual biobanks. Where there are examples of good practice and/or technical excellence, these should be advertised to the wider community. As an example, spreadsheets offer a particular challenge and should in nearly all circumstances be deemed wholly inappropriate for any biobank to use as a technical solution.

Before spreadsheets can be fully removed, however, there is a need to assess why they are being used and whether more advanced technical solutions exist. What spreadsheets do offer is the ability for a non-technical user to collect data and have full control over the data terminology, data structure, access rights (e.g., no firewall restrictions), and the ability to share data, even if that is simply e-mailing the spreadsheet. Therefore, it is necessary to fully understand why spreadsheets are used over other solutions and to understand how the ease of use, adaptability, and cost of existing systems leads biobanks to continue utilizing spreadsheets as the preferred solution.

The results of the first objective should feed into the development of an internationally accepted standard that specifies what is required as the default, in order to be classed as a biobanking software solution. As an example, API access should be a core requirement, rather than an optional and often costly extra. The second objective therefore is the development of an accredited list of database vendors who provide fit-for-purpose database solutions.

Finally, there is an argument for an increased role of ethical boards, governance, accreditation bodies, and funders to ensure that groups being authorized to collect samples have sufficient informatics capabilities to ensure the samples are used. Therefore, as well as assessing the capacity of the center to collect samples (such as consent and storage procedures), the collection of samples should only be authorized where the collectors can also demonstrate the technical capabilities to ensure the data surrounding the samples can be managed appropriately.

## Conclusion

The BCCTB was an ambitious project in the UK to bring together existing biobanks within a unified and harmonized network. The informatics system developed for the BCCTB network solved many of the technical challenges of creating such a network. There is a limit to what can be technically achieved if biobanks do not use appropriate technical solutions. Standardized access to clinical data provides a core challenge to BCCTB, and is one that remains challenging. There should be a full assessment on whether the necessary informatics capabilities exist both in the clinical environment from which the patient is consented and the biobank. Therefore, alongside the capturing of researchers needs in relation to samples, a technical assessment will ensure there is a full understanding about how an integrated network can be achieved, both in terms of the initial technical build of the system, but also the subsequent support required for the individual biobanks.

## References

[1] Thompson A, Brennon K, Cox A, et al. Evaluation of the current knowledge limitations in breast cancer research: A gap analysis. Breast Cancer Res 2008;10:R26 18371194 10.1186/bcr1983 PMC2397525

[2] Eccles S, Aboagye EO, Ali S, et al. Critical research gaps and translational priorities for the successful prevention and treatment of breast cancer. Breast Cancer Res 2013;15:R92 24286369 10.1186/bcr3493 PMC3907091

[3] Womack C, and Mager SR Human biological sample biobanking to support tissue biomarkers in pharmaceutical research and development. Methods 2014;70:3–11 24486552 10.1016/j.ymeth.2014.01.014

[4] Scott CT, Caulfield T, Borgelt E, Illes J Personal medicine–The new banking crisis. Nat Biotech 2012;30:141–147 10.1038/nbt.2116 22318029

[5] Lehmann S, Moore H, Ashton G, et al. International Society for Biological and Environmental Repositories (ISBER) Working Group on Biospecimen Science. Standard preanalytical coding for biospecimens: Review and implementation of the Sample PREanalytical Code (SPREC). Biopreserv Biobank 2012;10:366–374 24849886 10.1089/bio.2012.0012 PMC6463986

[6] Moore HM, Kelly A, McShane LM, Vaught J Biospecimen Reporting for Improved Study Quality (BRISQ). Transfusion 2013;53 10.1111/trf.12281 23844646

[7] International Society for Biological and Environmental Repositories, 2012 Best practices for repositories, collection, storage, retrieval, and distribution of biological materials for research. Biopreserv Biobank 2012;10:79–161 24844904 10.1089/bio.2012.1022

[8] Norlin L, Fransson MN, Eriksson M, et al. Biopreservation and biobanking, A minimum data set for sharing biobank samples, information, and data: MIABIS. Biopreserv Biobank 2012;10:343–348 24849882 10.1089/bio.2012.0003

[9] McShane LM, Altman DG, Sauerbrei W, et al. REporting recommendations for tumour MARKer prognostic studies (REMARK). Eur J Cancer 2005;41:1690–1696 16043346 10.1016/j.ejca.2005.03.032

[10] Simeon-Dubach D, Watson P Biobanking 3.0: Evidence based and customer focused biobanking. Clin Bioch 2014;47:300–308 10.1016/j.clinbiochem.2013.12.018 24406300

[11] Olson S, Berger A. Establishing Precompetitive Collaborations to Stimulate Genomics-Driven Drug Development: Workshop Summary. 2011: National Academies Press 21595119

[12] Fearn P, Michels C, Meagher K, Cada M 2012 International Society for Biological and Environmental Repositories Informatics Working Group: Survey results and conclusions. Biopreserv Biobank 2013;11:64–66 24845257 10.1089/bio.2012.1115

[13] Quality Information Committee. First National Data Quality Review: Executive Summary. 2010; Available from: http://www.england.nhs.uk/wp-content/uploads/2013/04/1ndqr-exec-sum.pdf Accessed 1442015

[14] Breast Cancer Campaign Tissue Bank. Available from: http://www.breastcancertissuebank.org Accessed 1442015

[15] Quinlan P, Ashfield A, Jordan L, et al. An integrated informatics platform to facilitate transforming tissue into knowledge. Breast Cancer Res 2010;12(Suppl 1): P27

[16] Angelow A, schmidt M, Weitmann K, et al. Methods and implementation of a central biosample and data management in a three-centre clinical study. Computer Methods Programs Biomed 2008;91:82–90 10.1016/j.cmpb.2008.02.002 18406002

[17] Winget MD, Baron JA, Spitz MR, et al. Development of common data elements: The experience of and recommendations from the early detection research network. Intl J Med Informatics 2003;70:41–48 10.1016/s1386-5056(03)00005-4 12706181

[18] Yuille M, van Ommen GJ, Brechot C, et al. Biobanking for Europe. Brief Bioinformatics 2008;9:14–24 17959611 10.1093/bib/bbm050

[19] Breast Cancer Campaign Tissue Bank SOP24. 2010 Available from: https://breastcancertissuebank.org/files/documentation/BCCTB-SOP-024%20Data%20Collection-4%20incl%20Oncologist%20list.pdf Last access on 129, 2015

[20] PostgreSQL. 2015; Available from: http://www.postgresql.org Last access on 129, 2015

[21] DataNucleus 2015. Available from: http://datanucleus.org Last access on 129, 2015

[22] Java. 2015. Available from: https://java.com/en/download/ Last access on 129, 2015

[23] Apache Tomcat. 2015. Available from: http://tomcat.apache.org Last access on 129, 2015

[24] Breast Cancer Campaign Tissue Bank search system. Available from: https://http://www.breastcancertissuebank.org/bcc/tissueBank?Name=search_main Accessed 1442015

[25] Cutts RJ, Guerra-Assuncao JA, Gadaleta E, Dayem Ulllah AZ, Chelala C BCCTBbp: The Breast Cancer Campaign Tissue Bank bioinformatics portal. Nucleic Acids Res 2015;43:D831–D836 25332396 10.1093/nar/gku984 PMC4384036

[26] STRATUM Work Packages 5 and 6. Register/Catalogue and Sample Characterisation Datasets. Available from: http://www.stratumbiobanking.org/docs/STRATUM Data and Register Final Report v1 0.pdf. Accessed 1442015

[27] Quinlan PR, Mistry G, Bullbeck H, Carter A; Confederation of Cancer Biobanks Working Group 3. A data standard for sourcing fit-for-purpose biological samples in an integrated virtual network of biobanks. Biopreserv Biobanking 2014;12:183–191 10.1089/bio.2013.0089 PMC4066222 24785371

[28] National Cancer Intelligence Network, Cancer and equality groups: Key metrics 2014 report. 2014

[29] van der Flier WM, Pijnenburg YA, Prins N, et al. Optimizing patient care and research: The Amsterdam Dementia Cohort. J Alzheimer's Dis 2014;41:313–327 24614907 10.3233/JAD-132306

[30] Chelsom JJ IGaywood I, Pande I Supporting Multiple Clinical Perspectives on a Patient-Centred Record Using Ontology Models. In: Workshops at the Twenty-Seventh AAAI Conference on Artificial Intelligence, Bellevue, Washington, 2013

